# Sex-specific differences in resting-state functional brain activity in pediatric concussion

**DOI:** 10.1038/s41598-023-30195-w

**Published:** 2023-02-25

**Authors:** Bhanu Sharma, Cameron Nowikow, Carol DeMatteo, Michael D. Noseworthy, Brian W. Timmons

**Affiliations:** 1 Child Health and Exercise Medicine Program, Department of Pediatrics, McMaster University, 1280 Main Street West, Hamilton, ON L8S4L8 Canada; 2 School of Rehabilitation Science, McMaster University, Hamilton, Canada; 3 CanChild Centre for Childhood Disability Research, McMaster University, Hamilton, Canada; 4 Imaging Research Centre, St. Joseph’s Healthcare, Hamilton, ON Canada; 5 Department of Electrical & Computer Engineering, McMaster University, Hamilton, Canada; 6 McMaster School of Biomedical Engineering, McMaster University, Hamilton, Canada; 7 Department of Radiology, McMaster University, Hamilton, Canada

**Keywords:** Neuroscience, Paediatric research

## Abstract

Pediatric concussion has a rising incidence and can lead to long-term symptoms in nearly 30% of children. Resting state functional magnetic resonance imaging (rs-fMRI) disturbances are a common pathological feature of pediatric concussion, though no studies have explicitly examined sex-differences with respect to this outcome, precluding a sex-specific understanding of the functional neuropathology of pediatric concussion. Therefore, we performed a secondary data analysis of rs-fMRI data collected on children with concussion (n = 29) recruited from in a pediatric hospital setting, with greater than 12:1 matched control data accessed from the open-source ABIDE-II database. Seed-based and region of interest (ROI) analyses were used to examine sex-based rs-fMRI differences; threshold-free cluster enhancement (TFCE) and a family-wise error (FWE) corrected p-values were used to identify significantly different clusters. In comparing females with concussion to healthy females, groupwise differences were observed irrespective of seed selected. Notably, we observed (in order of largest effect) hypo-connectivity between the anterior cingulate cortex of the salience network and the thalamus and precuneus (TFCE = 1473.5, p-FWE < 0.001) and the cingulate gyrus (TFCE = 769.3, p-FWE = 0.009), and the seed (posterior cingulate cortex (PCC)) of the default mode network and the paracingulate gyrus (TFCE = 1275.7, p-FWE  < 0.001), occipital pole right (TFCE = 1045.0, p-FWE = 0.001), and sub-callosal cortex (TFCE = 844.9, p-FWE = 0.005). Hyper-connectivity was observed between the salience network seed and the cerebellum (TFCE = 1719.3, p-FWE < 0.001) and the PCC and the thalamus (TFCE = 1198.3, p-FWE < 0.001), cuneal cortex (1070.9, p-FWE = 0.001), and lateral occipital cortex left (TFCE = 832.8, p-FWE = 0.006). ROI analyses showed 10 and 5 significant clusters of hypo- and hyper-connectivity in females, respectively. Only one cluster of difference was found between males with concussion and healthy males on seed-based analyses, and 3 clusters on ROI analyses. There are alterations in rs-fMRI in females with concussion at one-month post-injury that are minimally present in males, which provides further evidence that recovery timelines in pediatric concussion may differ by sex.

## Introduction

Concussion is a mild form of traumatic brain injury (TBI) that results in altered neurological function after biomechanical impact^1^. In pediatric populations, concussion is of particular concern given that it is one of the most common injuries among children and adolescents^2–4^ and has a rapidly rising incidence in those aged 10–19^5,6^. While the injury is transient for the majority of pediatric patients, between 14 and 29% experience persistent concussion symptoms^7–9^ (PCS; also referred to as post-concussion syndrome, marked by symptoms which last in excess of four weeks^1^). PCS can include somatic, cognitive, emotional, and sleep-related features that negatively impact academic outcomes^10^ and health-related quality of life^11,12^.

Brain function in pediatric concussion has been studied to understand the nature and extent of its impairment post-injury, as well as its potential etiological role with respect to concussion symptoms. Studies have measured brain function using resting-state functional magnetic resonance imaging (rs-fMRI), which maps regions of brain activity (by proxy of the blood-oxygen-level-dependent, or BOLD, response) and the relative associations between them in a task-independent manner. Pediatric rs-fMRI studies have shown increased functional connectivity in comparison to healthy controls in widely-studied brain networks within the first-week of injury^13–15^. At one-month post-injury (the expected time of recovery^1^), results of rs-fMRI studies are mixed^13,14,16–18^. With respect to studies of children diagnosed with PCS, one study found that within-network functional connectivity across seven validated brain networks did not differ between children with PCS (n = 110) vs. healthy peers (n = 20), although select PCS symptoms, sleep impairment, and poorer cognition were associated with connectivity in the concussed cohort^19,20^.

A notable limitation common to many pediatric concussion rs-fMRI studies are the imbalanced samples with respect to sex. Some studies involved male only cohorts^14,17,21^, whereas others had less than 25% female representation in their samples^13,18^; in some cases, data on sex were not reported^16,22^. Only a few studies had samples that approached balance (40–45% female) with respect to sex distribution^15,19,20,23^, though these studies did not stratify their results by sex, instead providing group-level data comparing mixed-sex cohorts of children with concussion to their healthy peers. The most direct data on sex-specific rs-fMRI differences come from a recent study involving adults with PCS^24^. More specifically, in this study, three commonly studied networks were examined through seed-based analyses, namely the default mode network (DMN), salience network, and fronto-parietal network; the authors reported reduced connectivity between the fronto-parietal network and nodes of the salience network in females with PCS.

The lack of a sex-specific understanding of rs-fMRI differences in pediatric concussion is a considerable knowledge gap, given that sex, as a biological variable, has been recognized as an understudied yet important consideration in neuroscience^25–27^. Further, a growing body of research demonstrates that concussion presents differently in boys vs. girls^28,29^. For example, a recent cohort study (n = 986) found that female adolescents with concussion endorse more symptoms on the 22-item and widely used SCAT5^30^ than concussed males, and are more likely to have a higher total symptom score^31^. Two large-scale, multi-center cohort studies have shown that females have a protracted recovery in comparison to males^28,29^, which align with other clinical data on disparate sex effects in concussion summarized in two recent systematic reviews^32,33^. Furthermore, differences in mechanism of injury between males and females, as well as factors that influence neurodevelopment (such as pubertal and hormonal status and genetics) can increase vulnerability to brain injury-induced pathologies in a sex specific manner (which are reviewed in detail by Arambula et al.)^64^. Therefore, we studied sex-specific rs-fMRI differences in pediatric concussion to address an important knowledge gap, and advance our understanding of how the functional neuropathology of concussion differs between males and females.

## Methods

### Design

The present study is a secondary analysis of data collected as part of two cohort studies (sharing recruitment methods, inclusion/exclusion criteria, and imaging parameters, as detailed below) on pediatric concussion. Control data were obtained from an open-source pediatric neuroimaging database (detailed below). This study was approved by the Hamilton Integrated Research Ethics Board (https://hireb.ca).

### Participants

Children (aged 9–17) experiencing concussion symptoms were recruited by the clinical study team from sites at or affiliated with McMaster University, including the McMaster Children’s Hospital and associated rehabilitation and sports medicine clinics, as well as through direct referral from community physicians. Children diagnosed with a concussion, and their families, were recruited for an intake assessment. Neuroimaging data were then collected as soon after recruitment as scheduling permitted. For the present study, exclusion criteria included: (i) more severe forms of head injury that required surgery, resuscitation, or admission to the critical care unit, (ii) complex injuries involving multiple organ systems, and (iii) diagnosed neurological disorder or developmental delay.

Imaging data on healthy children were acquired from the multi-site, internationally compiled, open-source Autism Brain Imaging Data Exchange II (ABIDE-II) database^34^. The ABIDE-II database is comprised of over one-thousand anonymized brains (including 557 healthy controls across age in our current study) collected from 19 sites, primarily in North America and continental Europe, yielding nearly 75 publications to date. Both anatomical and functional scans from the ABIDE-II database were pulled to serve as ~ 12:1 age- and sex-matched typically developing controls for our participants with concussion. Specific matching criteria were not applied when selecting controls. Rather, all children within the age range of interest (9–17) were retrieved as potential controls. While the hardware (with respect to make of the scanners, and the type of head coil used, although all scanners were 3.0 T) varied slightly between sites that participated in the ABIDE initiative, quality control data are indicative of homogeneity in data (with respect to signal-to-noise ratio, data smoothness, number of outlier scans) across sites^34^. Imaging parameters and scanner make and models can be found in Supplemental Table 1.

### MRI procedures data acquisition

All children with concussion were scanned at a single site (Imaging Research Centre [IRC] at St. Joseph’s Healthcare, Hamilton) using a 3-Tesla GE Discovery MR750 MRI scanner and a 32-channel phased array head receiver coil. Upon entering the IRC, participants (as well as their parents and/or guardians if aged 16 years or younger) were led through an intake questionnaire by the MRI technologist, who then situated the participant in the MRI, using foam cushioning to minimize discomfort and motion during the scan. The MRI technologist remained in verbal contact with the participant via intercom throughout the scan.

With respect to MRI data collection, first, a 3-plane localizer sequences was acquired. Anatomical images were then collected using a 3D inversion recovery-prepped fast SPGR T1-weighted sequence (TR/TE = 11.36/4.25 ms, TI = 450mms, flip angle = 12°, 512 × 256 matrix interpolated to 512 × 512, 22 cm axial FOV, 1 mm thick). Resting state fMRI (rs-fMRI) involved BOLD imaging (gradient echo EPI, TR/TE = 2000/35 ms, flip angle = 90°, 64 × 64 matrix, 180 time points, 3 mm thick, 22 cm FOV), wherein participants were asked to remain awake, keep their eyes open, and not to think of anything in particular. A B_0_ map was acquired for resting state scans, using the same geometric prescription as the rs-fMRI scan. A B_o_ mapping tool available on the GE scanner provided a parametric map of field homogeneity in Hz. In regards to the scanning sequence, the rs-fMRI data were acquired within 10-min of entering the MRI, as to avoid motion onset by restlessness later in scans as we have observed in this population. Additional data were collected (including DTI^35^ and task-based fMRI data^36^) as part of the imaging battery, but are not relevant to the present study.

With respect to control data from the ABIDE-II database, only scans with a minimum of 180 time-points were used as age- and sex-matched controls. B_0_ data were not available for healthy controls. Approximately 10% of the ABIDE-II data did not meet our quality control processes (as implemented in CONN 21a, as below) and were ultimately discarded from the analysis.

### MRI pre-processing and analyses

Pre-processing of imaging data was performed in CONN 21a^37^ (which draws on some the functionality of SPM12^38^), run on MATLAB R2021b. For concussion data only, given that B_0_ maps were not available for controls, unwarping of functional data was performed outside of CONN using the *epiunwarp* script^39^. Fieldmaps were not available for all controls, and were thus only applied to the concussion group (to ensure our data were as rigorously pre-processed prior to comparison to the larger control group). While it would be ideal to apply fieldmap corrections to all participants, we were unable to do so given the lack of such data on controls. However, spatial smoothing (per the prescription below) is likely to make the effects of fieldmap correction minimal. Per our quality assurance, the timeseries of the fieldmap corrected and uncorrected data (for the concussion group) were similar (Supplemental Fig. 1), and statistical tests revealed no differences between the corrected and uncorrected data.

Unwarped images were inputted into the pre-processing pipeline which involved the following steps: (1) Functional realignment with co-registration to the first acquired image^40^; (2) Slice-timing correction to the mid-point of each TR^41^; (3) Functional data outlier detection using SPM’s Artifact Detection Tool (ART)^38^; (4) Direct segmentation and normalization/registration of functional data to MNI space (1 mm and 2 mm isotropic voxels for anatomical and functional data, respectively), based on posterior tissue probability maps; and (5) spatial smoothing of functional data with a Gaussian kernel of full-width at half-maximum (FWHM) of 6 mm. All data were inspected visually after pre-processing, as well as by running CONN’s quality assurance assessment tool.

Next, de-noising procedures were performed in CONN. First, CONN’s anatomical component-based noise correction procedure (*aCompCorr*) was used to project out noise components (associated with cerebral white matter and cerebrospinal regions^42^, outlier scans^43^, and subject motion^44^). Subsequently, temporal filtering was performed, filtering out frequencies below 0.008 Hz and above 0.1 Hz. Data were again inspected visually and per the quality assurance metrics offered by CONN.

Seed-based connectivity and ROI-to-ROI based connectivity measures were then computed for each individual subject. Seed regions from four, large-scale, validated and clinically-salient (in pediatric concussion and otherwise) resting-state brain networks were used^45–48^. These included the DMN (seeded at the posterior cingulate cortex [1, − 61, 38]), salience network (SN, seeded at the anterior cingulate cortex [0, 22, 35]), fronto-parietal network (FPN, seeded at the lateral pre-frontal cortices), and sensorimotor network (SMN, seeded superiorly at the pre-central gyrus [0, − 31, 67]). Given that this study is the first to look at sex-differences in rs-fMRI in pediatric concussion, seed-based analyses were employed to explore the relation between these seeds and all other voxels of the brain; an accompanying ROI-to-ROI analysis was also performed (which examines the associations between 164 regions defined by the Harvard–Oxford atlas).

Cluster-level inferences were made per Threshold Free Cluster Enhancement (TFCE)^49^, which avoids the use of an a priori cluster-forming height threshold. For each groupwise contrast (as specified above) permutation tests (involving 1000 permutations) were used to derive a null distribution that the observed effects were then compared to, and a TFCE score associated with family-wise error (FWE) corrected p-value for each cluster was obtained. Through these permutations, the expected distribution of TFCE scores under the null hypothesis is estimated. Then, at each voxel, the observed TFCE score is compared to the permuted null distribution, and each voxel/cluster is given a TFCE score. Only those observed voxels/clusters that survive comparison to the permuted null distribution are reported. This method is conservative and reduces family-wise error, which also speaks to the robustness of our findings/analysis. Further, TFCE has the advantage of being associated with a lower false-positive rate than traditional cluster-size tests based on random field theory^50^.

Between-group contrasts were set up in CONN 21a, controlling for the effects of age. More specifically, we performed the following between-group analyses: All Concussion vs. All Controls, Female_Concussion_ vs. Female_Control_, Male_Concussion_ vs. Male_Control_, and a 2 × 2 (Group × Sex) ANCOVA. For each contrast and for each seed-region, significantly different clusters were identified using TFCE. The effect sizes associated with each significant cluster were computed, along with a t-score to statistically compare effect size differences at the cluster level between groups.

## Results

Demographic and injury data of the 29 children with concussion and 361 controls are summarized in Table 1. Age and sex distribution did significantly differ between cohorts; however, we controlled for age in all analyses and performed both mixed-sex cohort analyses as well as single-sex (i.e., healthy female vs. female with concussion) analyses. Within the concussion group, males and females had similar PCSS scores (47.8 vs. 41.6, p = 0.511) at time of imaging per an independent samples t-test. Patients with concussion were, on average, approximately one-month post-injury (28.8 ± 14.5 days) at time of imaging, and had no history of anxiety, depression, sleep disorder, or psychiatric diagnosis.

**Table 1:** Demographic and injury-related variables.

	Concussion (n = 29)	Healthy (n = 361)	Significance
Age	14.2 (2.5)	11.0 (2.2)	p < 0.05
Male	13.8 (2.7)	10.9 (2.2)	p < 0.05
Female	14.8 (2.3)	11.4 (2.4)	p < 0.05
% female	55.2%	33.2%	p < 0.05
Time-post injury	28.5 (16.5)	–	–
Male	24.2 (14.2)		
Female	34.1 (21.9)		
Previous concussion	0, n = 17	–	–
	1, n = 8		
	2, n = 2		
Male	0, n = 9		
	1, n = 3		
	2, n = 1		
Female	0, n = 10		
	1, n = 5		
	2, n = 1		
Mechanism of injury	70.3% sport	–	–
	22.2% non-sport related falls		
	7.4% motor vehicle collision		

The following figures depict the five clusters of greatest change (as per TFCE scores and associated p-values), per network and per analysis. A full list of significant clusters can be found in Supplemental Table 2.

### Concussion vs. control group comparison

**Figure 1:**
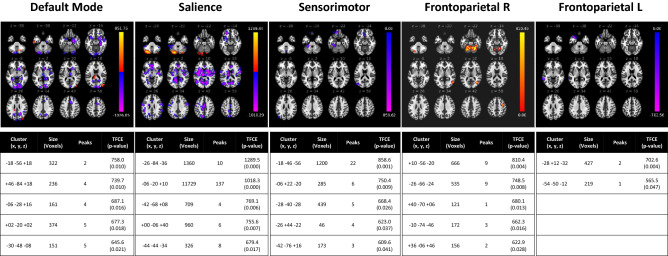
rs-fMRI differences between children with concussion and their healthy peers (mixed-sex cohorts). Clusters (x, y, z) denote standard MNI coordinates at the center of cluster mass, and size represents number of voxels. Up to five clusters with the largest TFCE scores that survived a p-FWE < 0.05 per TFCE are displayed; see Supplemental Table 2 for all clusters that survived analysis.

In the concussion cohort, there was significantly reduced functional connectivity (all p-FWE < 0.05, with corresponding TFCE scores in Fig. 1) between the seed-region of the: (i) DMN and the hippocampus, amygdala, and caudate (right), precuneous cortex, paracingulate gyrus (right), occipital cortex, and precuneous cortex; (ii) SMN and the cerebellum (left), frontal pole (left), temporal fusiform cortex (left), lateral occipital cortex (left); (iii) SA and the precuneous cortex, lateral occipital cortex (left), cingulate gyrus (left), cerebellum, and precentral gyrus (left); FPN-L and the temporal pole (left), and middle temporal gyrus (left). Further, there was increased functional connectivity between the seed-region of the: (i) DMN and cerebellum, lateral occipital cortex (right); SA and the cerebellum; FPN R and the cerebellum, lateral occipital cortex (right), precentral gyrus (right). These data are depicted in Fig. 1.

**Figure 2:**
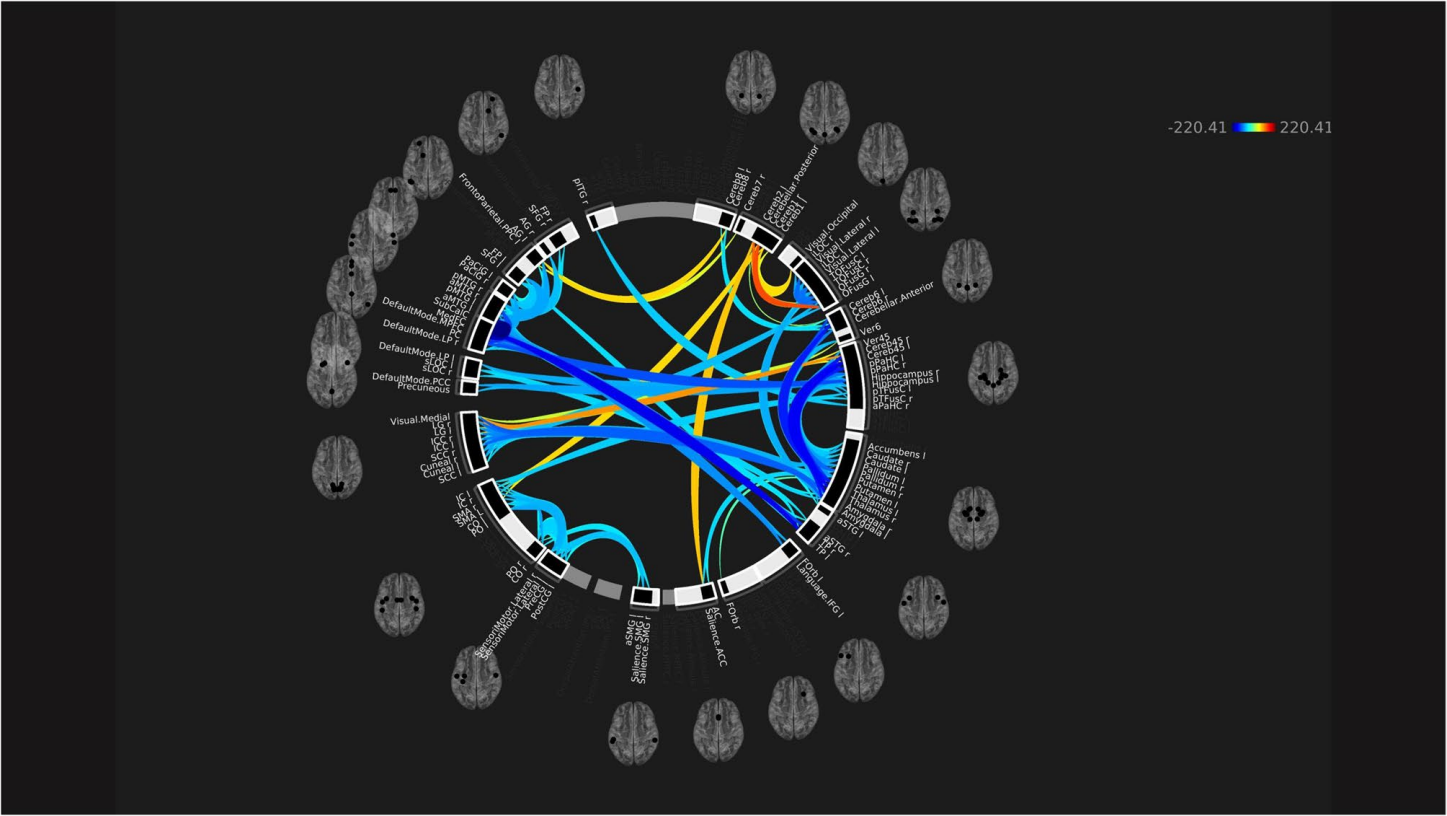
Significantly increased (warm colours) and decreased (cool colours) ROI-to-ROI connectivity in children with concussion in comparison to controls (mixed-sex cohort).

At the ROI-to-ROI level, there was a broad pattern of decreased functional connectivity between multiple brain regions bilaterally, and fewer instances of increased connectivity between pairs of ROIs. The clusters of ROIs with significantly reduced connectivity included: regions within the default mode network itself; the default mode network and the hippocampi; the cerebellum and the amygdala, putamen, and thalamus (see Fig. 2).

### Healthy males vs. males with concussion

**Figure 3:**
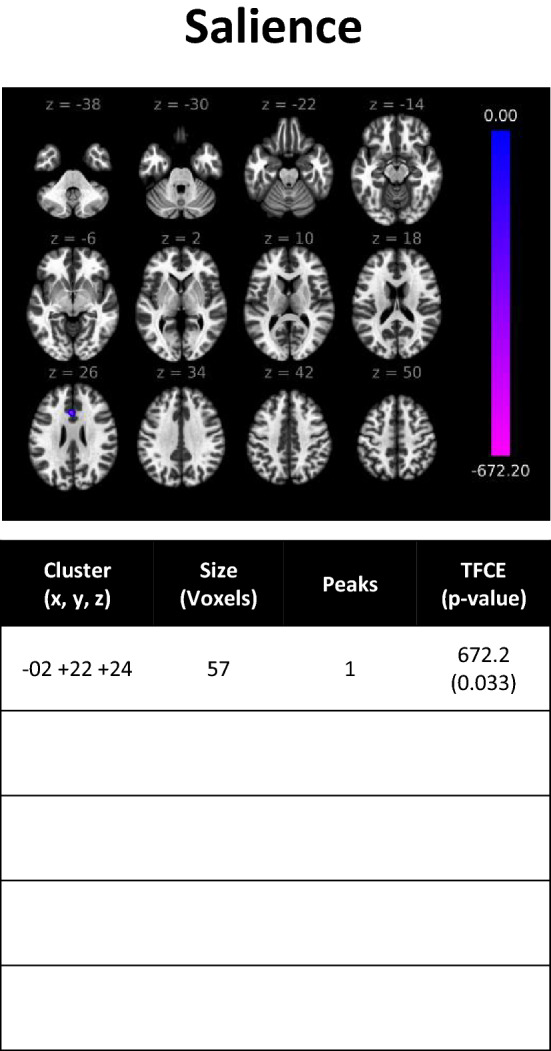
rs-fMRI differences between males with concussion and healthy males. Clusters (x, y, z) denote standard MNI coordinates at the center of cluster mass, and size represents number of voxels. Only one cluster survived analysis.

**Figure 4:**
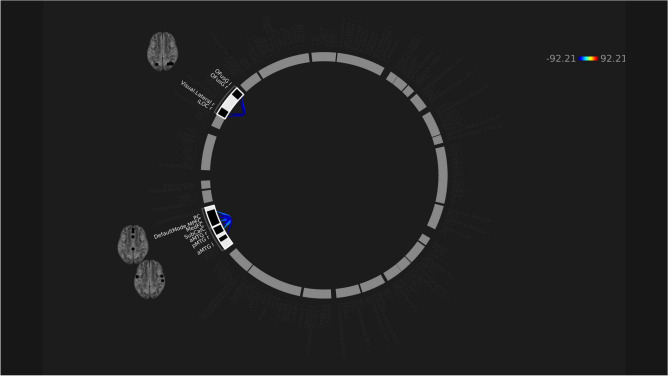
Significantly decreased (cool colours) ROI-to-ROI connectivity in males with concussion in comparison to healthy male controls.

Per seed-based analyses, hypoconnectivity was observed between the seed of the salience network and a small voxel of clusters in the cingulate gyrus (Fig. 3). ROI-to-ROI analyses showed 3 clusters of hypoconnectivity between frontal and medial brain structures, as well as within the occipital lobe (Fig. 4).

### Healthy females vs. females with concussion

**Figure 5:**
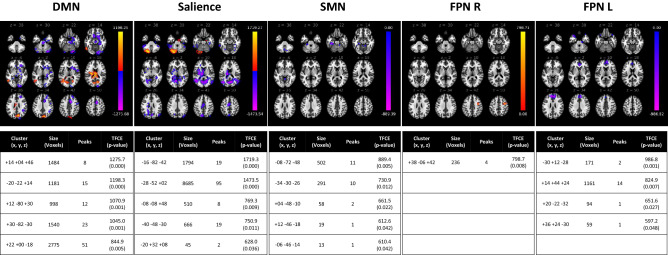
rs-fMRI differences between females with concussion and healthy females. Clusters (x, y, z) denote standard MNI coordinates at the center of cluster mass, and size represents number of voxels. Only one cluster survived analysis for the frontoparietal right; with multiple clusters identified for all other seeded networks.

In females, with respect to the DMN, there was increased connectivity between the DMN seed and parts of the cuneal cortex (right), caudate, and thalamus, and reduced connectivity between said seed and primarily the paracingulate gyrus (right), occipital pole (right), hippocampus (right), and precentral gyrus (right). Hypoconnectivity was observed between the SMN seed and the cerebellum, parahippocampal gyrus (left), and vermis. The seed region of the SA had reduced connectivity with regions including the thalamus, cingulate gyrus, and cerebellum. Further, the FPN R was associated with increased functional connectivity with the precentral gyrus, and the FPN L was associated with reduced connectivity in the temporal pole (left), paracingulate gyrus (bilaterally) and superior frontal gyrus (bilaterally); see Fig. 5.

**Figure 6:**
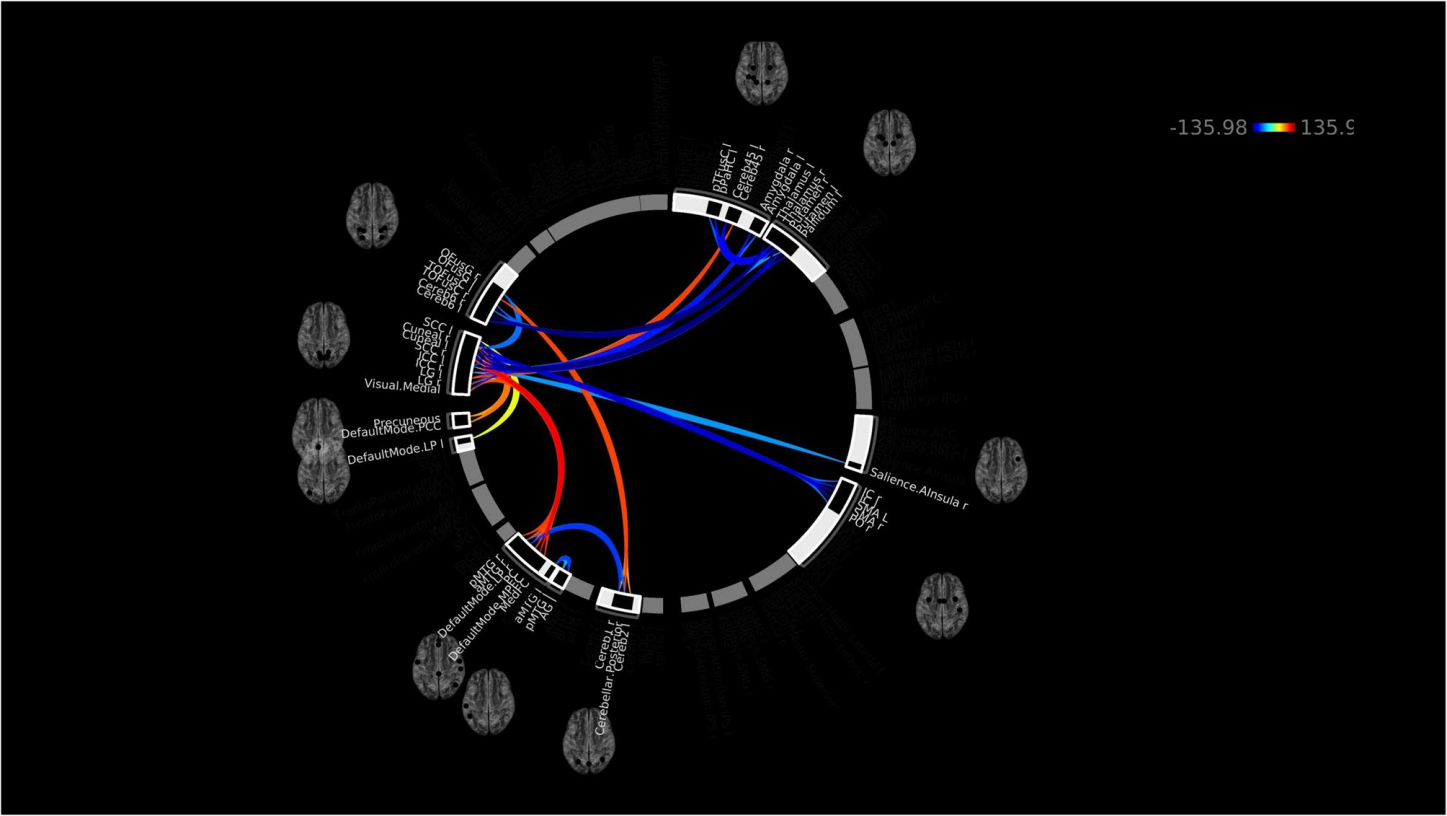
Significantly increased (warm colours) and decreased (cool colours) ROI-to-ROI connectivity in females with concussion in comparison to healthy females.

In females, groupwise ROI-to-ROI analyses showed that there was increased connectivity between ROIs in the cuneal cortex and cerebellum, as well as between the cuneal cortex and default mode network. There was also reduced connectivity between temporal brain structures and those in the occipital region in females with concussion compared to healthy females (Fig. 6).

Per the ANCOVA, seed-based analyses identified one cluster of hyperconnectivity in the paracingulate gyrus relative to the FPN L seed (TFCE = 863.4, p-FWE = 0.002). No differences were observed per the ANCOVA analyses on ROI-to-ROI metrics.

## Discussion

Our study is the first to report that in pediatric concussion, there are rs-fMRI disturbances observed in females that are not present in the males. To date, the majority of studies on rs-fMRI in pediatric concussion have either not studied females or had a small female representation (< 25%) in their clinical samples. Our findings, therefore, provide the first insight into functional disturbances in pediatric concussion by sex.

In pediatric concussion, studies have attempted to link rs-fMRI disturbance to symptoms but have not found a clear relationship^14,16,23^. This may in part be attributable to symptom and rs-fMRI data not being disaggregated by sex in prior studies that have studied both of these measures. Our results show that following concussion, at approximately one-month post-injury, there are alterations in rs-fMRI activity in females (in comparison to their healthy age- and sex-matched peers) that are not observed in males. Symptom studies align with this, reporting sex-differences with respect to symptoms in pediatric concussion^28,29,31–33^. With a large evidence-base suggesting that symptom presentation differs in pediatric concussion by sex, and with the current study demonstrating sex-based rs-fMRI differences in children with concussion, there is reason to hypothesize that this variable symptom presentation has an underlying functional sex-specific neuropathology. Past studies that did not find a clear relationship between concussion symptoms and functional brain pathology may not have observed such an effect because their analyses were not stratified by sex, which (as shown in the present study) provides insights that mixed-sex analyses do not. Future studies should collect data on symptoms and rs-fMRI and stratify analyses by sex to better understand the relationship between these variables.

Past studies that have identified differences in rs-fMRI activity between controls and those with a brain injury have been conducted in primarily male samples^13,14,17,18,21^. Therefore, these studies reported rs-fMRI differences that, with respect to percent representation within the sample, were largely driven by males. These findings do not reconcile with ours, given that we found minimal differences between healthy males and males with concussion with respect to their resting state fMRI activity. Further, in our study, we found differences when comparing females with concussion to healthy females that were otherwise masked when performing a 2 × 2 (Group × Sex) ANCOVA. Our findings, however, do converge with data on adults with PCS, where there were marked differences between healthy females and females with concussion^24^. With respect to pre-clinical research, animal studies are also discrepant with respect to sex effects of brain injury on, for example, brain morphology and functional outcomes in adolescent rodents^64^. Collectively, these data suggest that additional research is needed to understand how the neuropathology of concussion varies by sex.

Data on the risk of secondary concussion by sex are limited, with the majority of studies to date focusing on predominantly male samples^51^. Our study, however, shows that in females, concussion can impact regions of the brain including the insular cortex, cuneal cortex, and thalamus, which are involved in processing of sensory and/or visual information as well as motor control^52^. While data on whether brain function remains impaired at medical clearance to return to activity are mixed^53,54^, our findings (wherein imaging was performed, on average, at the time when clinical recovery from concussion is expected to occur) suggest that functional brain impairments persist in females but not males. This suggests that resolution of functional pathologies in sensory and motor areas of the brain may be sex-dependent, and that return-to-sport guidelines stand to be informed by sex-specific data.

A recent review on sex differences in concussion (pediatric and adult) identified that injury may lead to alterations in the hypothalamic-pituitary-ovarian axis, and subsequent hormonal fluctuations that may be responsible for the more severe symptoms in females^55^. rs-fMRI studies in other endocrinological populations demonstrate that alterations in functional brain activity are associated with abnormal hormonal responses^56–59^. With our study pointing to rs-fMRI disturbances in females with concussion that are not present in males, these functional brain changes may mediate or be related to a variable hormonal response that has an ultimate impact on the female concussion symptomology. Research that directly examines the relationship between brain activity, hormonal fluctuations, and symptomatology is required to build on this possibility.

Large-scale studies (such as the Philadelphia Neurodevelopmental Cohort [PNC], which included nearly 1600 imaging assessments on those aged 8 to 21 years^60,61^) have shown that rs-fMRI patterns vary by sex throughout neurodevelopment. Other studies have also demonstrated sex differences with respect to functional brain activity (and brain morphology, more broadly), and that these differences relate to variable neurodevelopment of networks such as the DMN^62^. In our analyses, we compared concussed males and females to their respective age- and sex-matched control groups, thereby avoiding the potential confounding neurodevelopmental effects that may arise when comparing males to females directly.

## Limitations and future directions

Future studies should include longitudinal assessments to determine if sex-differences in rs-fMRI activity in pediatric concussion vary from acute to the chronic stages of injury. This line of research, combined with existing evidence on pediatric symptom trajectories post-concussion, would help in understanding whether there is a functional neuropathology driving the symptom response longitudinally in children who have delayed recoveries. These studies should also perform rs-fMRI assessments in children who are asymptomatic, given that the broader literature has shown that neurophysiological disturbances can outlast symptoms^63^; understanding whether functional neuropathology outlasts clinical recovery can improve our understanding of the vulnerability of the brain to secondary injuries in a sex-specific manner. Moreover, additional experimental control in prospective research may help us better understand rs-fMRI changes in pediatric concussion. Specifically, pubertal status has a known impact on neurodevelopment, and we were unable to control for these effects in this secondary data analysis. Future research should consider measuring pubertal status (using the Tanner Stages, for example) and understanding its effects on resting state brain activity in pediatric concussion. Studying interactions between sex and pubertal status may also offer additional insight into rs-fMRI following pediatric concussion.

## Conclusions

This is the first study to explicitly study and report on sex-specific rs-fMRI differences in pediatric concussion. At one-month post-injury, we report on differences in females with concussion (in comparison to their healthy peers) that are not apparent in males. This research further speaks to the need for more sex-specific analyses in concussion research.

## Supplementary Information

Supplementary Table 1.

Supplementary Table 2.

Supplementary Legends.

Supplementary Figure 1.

## Data Availability

The datasets used and/or analysed during the current study available from the corresponding author on reasonable request.
