# Supplementary material for: Sex-specific differences in resting-state functional brain activity in pediatric concussion
Source: Sci Rep. 2023 Feb 25;13:3284. doi: 10.1038/s41598-023-30195-w (PMC9968337; doi:10.1038/s41598-023-30195-w)
Supplement: Supplementary file 1 — Supplementary Table 1. [file 41598_2023_30195_MOESM1_ESM.docx]

**Supplemental Table 1.** Scan parameters for open-source ABIDE-II data.

|  |  | **Anatomical 3D T1** | **Functional** | | | | |
| --- | --- | --- | --- | --- | --- | --- | --- |
| **Site** | **n** | **inversion time (ms)** | **TE**  **(ms)** | **TR**  **(ms)** | **Matrix** | **FOV**  **(mm)** | **Vendor** |
| Barrow Neurological Institute | 2 | 900 | 25 | 3000 | 64x64 | 240x240 | Philips Ingenia (3.0T) |
| Erasmus University Medical Center Rotterdam | 21 | 350 | 30 | 2000 | 64x64 | 230x230 | GE MR750 (3.0T) |
| ETH Zurich | 3 | 1150 | 25 | 2000 | 80x77 | 240x240 | Philips Achieva (3.0T) |
| Georgetown University | 47 | 1100 | 30 | 2000 | 64x64 | 192x192 | Siemens TriTim (3.0T) |
| Kennedy Kreiger Institute | 144 | 900 | 30 | 2500 | 84x81 | 256x256 | Philips Achieva (3.0T) |
| NYU Langmore Medical Centre | 14 | 1100 | 30 | 2000 | 80x80 | 240x240 | Siemens Allegra (3.0T) |
| Oregon Health and Science University | 44 | 900 | 30 | 2500 | 64x64 | 240x240 | Siemens TriTim (3.0T) |
| Trinity Centre for Health Sciences | 11 | 1150 | 27 | 2000 | 80x80 | 240x240 | Philips Achieva (3.0T) |
| San Diego State University | 21 | 600 | 30 | 2000 | 64x64 | 220x220 | GE MR750 (3.0T) |
| Stanford University | 20 | 1000 | 30 | 2000 | 64x64 | 240x240 | GE SIGNA (3.0T) |
| University of California Davis | 14 | 1050 | 24 | 2000 | 64x64 | 224x224 | Siemens TriTim (3.0T) |
| University of California Los Angeles | 8 | 853 | 28 | 3000 | 64x64 | 192x192 | Siemens TriTim (3.0T) |
| University of Miami | 9 | 650 | 30 | 2000 | 64x64 | 240x240 | GE Healthcare (3.0T) |
| University of Utah School of Medicine | 3 | 900 | 28 | 2000 | 64x64 | 220x220 | Siemens TriTim (3.0T) |
