# Supplementary material for: Sex-specific differences in resting-state functional brain activity in pediatric concussion
Source: Sci Rep. 2023 Feb 25;13:3284. doi: 10.1038/s41598-023-30195-w (PMC9968337; doi:10.1038/s41598-023-30195-w)
Supplement: Supplementary file 2 — Supplementary Table 2. [file 41598_2023_30195_MOESM2_ESM.docx]

**Supplemental Table 2:** All significant clusters identified through our analyses.

| CONTRAST: Healthy vs. Concussion (Mixed-sex cohorts) | | | | | | | |
| --- | --- | --- | --- | --- | --- | --- | --- |
|  | **Cluster** | | | **Size (Voxels)** | **Peaks** | **TFCE score** | **p-value** |
|  | **x** | **y** | **z** |  |  |  |  |
| DMN | -18 | -56 | 18 | 322 | 2 | 758.03 | 0.01 |
|  | 46 | -84 | 18 | 236 | 4 | 739.65 | 0.011 |
|  | -6 | -28 | 16 | 161 | 4 | 687.06 | 0.016 |
|  | 2 | -20 | 2 | 374 | 5 | 677.29 | 0.018 |
|  | -30 | -48 | -8 | 151 | 5 | 645.61 | 0.021 |
|  | 42 | -46 | 26 | 66 | 2 | 596.33 | 0.033 |
|  | 24 | 32 | -10 | 225 | 2 | 589.95 | 0.034 |
|  | -26 | 4 | -18 | 321 | 3 | 577.93 | 0.039 |
|  | -56 | -20 | -30 | 93 | 1 | 577.3 | 0.039 |
|  | -46 | -38 | -28 | 58 | 1 | 561.64 | 0.046 |
|  | 22 | 34 | 38 | 49 | 2 | 558.07 | 0.047 |
| SMN | -18 | -46 | -56 | 1200 | 22 | 858.62 | 0.001 |
|  | -6 | 22 | -20 | 285 | 6 | 750.4 | 0.009 |
|  | -28 | -40 | -28 | 439 | 5 | 668.39 | 0.026 |
|  | -26 | 44 | -22 | 46 | 4 | 623.04 | 0.037 |
|  | -42 | -76 | 16 | 173 | 3 | 609.55 | 0.041 |
|  | -34 | 52 | -14 | 8 | 1 | 598.8 | 0.045 |
| SALIENCE | -26 | -84 | -36 | 1360 | 10 | 1289.44 | 0.000 |
|  | -6 | -20 | 10 | 11729 | 137 | 1018.29 | 0.000 |
|  | -42 | -68 | 8 | 709 | 4 | 769.09 | 0.006 |
|  | 0 | -6 | 40 | 960 | 6 | 755.58 | 0.007 |
|  | -44 | -44 | -34 | 326 | 8 | 679.35 | 0.017 |
|  | -60 | -8 | 14 | 638 | 4 | 672.46 | 0.018 |
|  | 62 | 18 | 12 | 196 | 2 | 631.59 | 0.030 |
|  | 58 | -46 | 20 | 135 | 1 | 605.67 | 0.036 |
|  | 34 | -84 | -32 | 84 | 4 | 602.96 | 0.039 |
|  | -32 | 14 | 36 | 125 | 8 | 594.1 | 0.040 |
|  | 44 | -54 | -38 | 131 | 1 | 589.4 | 0.043 |
|  | -28 | -2 | 38 | 46 | 1 | 589.04 | 0.043 |
|  | 54 | -72 | 20 | 105 | 3 | 588.66 | 0.043 |
|  | 14 | -88 | -26 | 16 | 1 | 572.72 | 0.047 |
|  | 38 | -86 | 30 | 32 | 1 | 569.53 | 0.049 |
|  | -20 | -16 | 40 | 17 | 1 | 567.5 | 0.050 |
| FPN R | 10 | -56 | -20 | 666 | 9 | 810.4 | 0.004 |
|  | -26 | -66 | -24 | 535 | 9 | 748.54 | 0.008 |
|  | 40 | -70 | 6 | 121 | 1 | 680.07 | 0.013 |
|  | -10 | -74 | -46 | 172 | 3 | 662.32 | 0.016 |
|  | 36 | -6 | 46 | 156 | 2 | 622.9 | 0.028 |
|  | 52 | -66 | -2 | 115 | 2 | 575.88 | 0.042 |
|  | 22 | -24 | -16 | 4 | 1 | 559.34 | 0.048 |
| FPN L | -28 | 12 | -32 | 437 | 2 | 702.56 | 0.004 |
|  | -54 | -50 | -12 | 219 | 1 | 565.48 | 0.047 |
| CONTRAST: Healthy female vs. female with concussion | | | | | | | |
| DMN | 14 | 4 | 46 | 1484 | 8 | 1275.68 | 0.000 |
|  | -20 | -22 | 14 | 1181 | 15 | 1198.25 | 0.000 |
|  | 12 | -80 | 30 | 998 | 12 | 1070.91 | 0.001 |
|  | 30 | -82 | -30 | 1540 | 23 | 1044.99 | 0.001 |
|  | 22 | 0 | -18 | 2775 | 51 | 844.9 | 0.005 |
|  | -58 | -64 | -12 | 541 | 10 | 832.78 | 0.006 |
|  | 56 | -2 | 8 | 146 | 3 | 702.66 | 0.018 |
|  | 42 | -44 | 26 | 269 | 2 | 697.33 | 0.019 |
|  | 26 | 40 | 26 | 377 | 5 | 685.17 | 0.022 |
|  | 24 | -68 | -52 | 127 | 1 | 684.43 | 0.022 |
|  | -34 | -86 | -32 | 226 | 5 | 681.2 | 0.022 |
|  | 46 | -4 | 34 | 134 | 5 | 654.42 | 0.031 |
|  | -30 | -54 | -10 | 72 | 2 | 643.87 | 0.033 |
|  | 50 | -10 | 0 | 68 | 3 | 641.51 | 0.035 |
|  | 10 | -4 | -30 | 33 | 2 | 626.9 | 0.038 |
|  | 20 | -78 | -4 | 46 | 1 | 626.15 | 0.038 |
|  | 8 | -20 | -6 | 24 | 2 | 614.61 | 0.042 |
|  | 2 | -32 | -8 | 3 | 1 | 600.79 | 0.047 |
|  | -4 | -30 | -6 | 3 | 1 | 599.87 | 0.048 |
| SMN | -8 | -72 | -48 | 502 | 11 | 889.39 | 0.005 |
|  | -34 | -30 | -26 | 291 | 10 | 730.91 | 0.012 |
|  | 4 | -48 | -10 | 58 | 2 | 661.52 | 0.022 |
|  | 12 | -46 | -18 | 19 | 1 | 612.59 | 0.042 |
|  | -6 | -46 | -14 | 13 | 1 | 610.35 | 0.042 |
|  | 18 | -42 | -26 | 25 | 1 | 608.72 | 0.042 |
|  | -38 | -44 | -14 | 18 | 1 | 596.36 | 0.047 |
| SALIENCE | -16 | -82 | -42 | 1794 | 19 | 1719.27 | 0.000 |
|  | -28 | -52 | 2 | 8685 | 95 | 1473.54 | 0.000 |
|  | -8 | -8 | 48 | 510 | 8 | 769.34 | 0.009 |
|  | -40 | -48 | -30 | 666 | 19 | 750.87 | 0.011 |
|  | -20 | 32 | 8 | 45 | 2 | 628.01 | 0.036 |
|  | -36 | -8 | 60 | 54 | 2 | 623.39 | 0.037 |
|  | -62 | -46 | -16 | 47 | 1 | 616.66 | 0.039 |
|  | 34 | -60 | -18 | 19 | 1 | 610.46 | 0.042 |
|  | -28 | -6 | 66 | 79 | 1 | 609.79 | 0.042 |
|  | 38 | -66 | -24 | 30 | 1 | 605.38 | 0.045 |
|  | 32 | -76 | -14 | 16 | 1 | 601.91 | 0.048 |
|  | -62 | -66 | 8 | 1 | 1 | 599.97 | 0.049 |
|  | 12 | -12 | -32 | 95 | 1 | 599.84 | 0.049 |
| FPN R | 38 | -6 | 42 | 236 | 4 | 798.71 | 0.008 |
| FPN L | -30 | 12 | -28 | 171 | 2 | 986.82 | 0.001 |
|  | 14 | 44 | 24 | 1161 | 14 | 824.86 | 0.007 |
|  | 20 | -22 | -32 | 94 | 1 | 651.6 | 0.027 |
|  | 36 | 24 | -30 | 59 | 1 | 597.15 | 0.048 |
| CONTAST: Healthy males vs. males with concussion | | | | | | | |
| SALIENCE | -2 | 22 | 24 | 57 | 1 | 672.2 | 0.033 |
| CONTRAST: 2x2 (Group x Sex) ANCOVA | | | | | | | |
| FPN L | -10 | +42 | +26 | 995 | 13 | 873.4 | 0.002 |
