# Supplementary material for: Sex-specific differences in resting-state functional brain activity in pediatric concussion
Source: Sci Rep. 2023 Feb 25;13:3284. doi: 10.1038/s41598-023-30195-w (PMC9968337; doi:10.1038/s41598-023-30195-w)
Supplement: Supplementary file 3 — Supplementary Legends. [file 41598_2023_30195_MOESM3_ESM.docx]

**Supplemental figure 1:** The fieldmap corrected and uncorrected timeseries for the concussion group. For this analysis, we used data only from the concussion group. We derived the timeseries with and without fieldmap correction (for each network studied). The timeseries are overlaid in this figure, demonstrating their similarity. Further statistical testing (namely t-tests to compare patterns of activity, performed within CONN) revealed no differences between the fieldmap corrected and uncorrected data.
