# Supplementary figures and images for: Sex-specific differences in resting-state functional brain activity in pediatric concussion
Source: Sci Rep. 2023 Feb 25;13:3284. doi: 10.1038/s41598-023-30195-w (PMC9968337; doi:10.1038/s41598-023-30195-w)

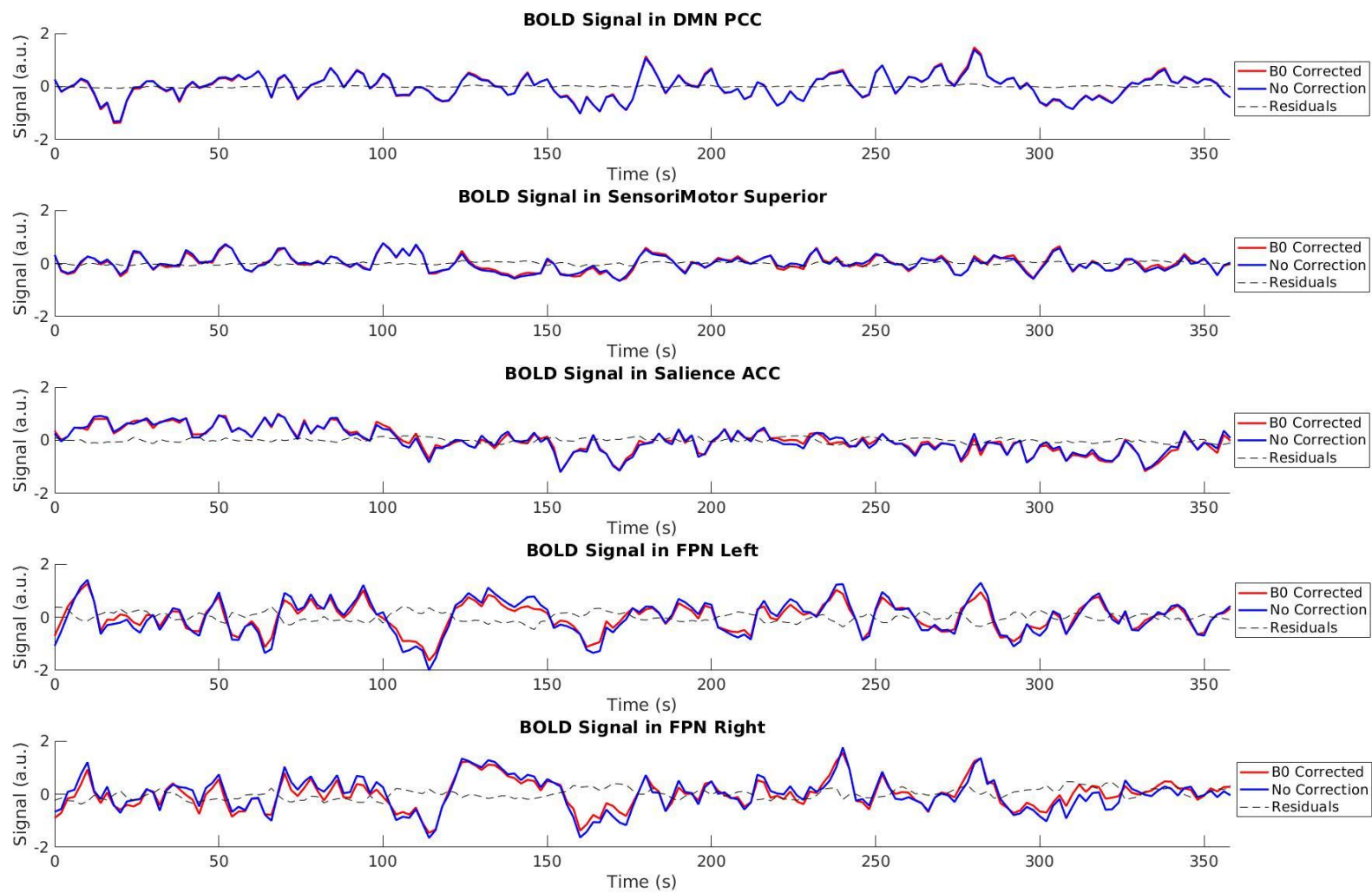

Supplement: Supplementary file 4 — Supplementary Figure 1. [file 41598_2023_30195_MOESM4_ESM.pdf]
